# Simplified risk stratification criteria for identification of patients with MRSA bacteremia at low risk of infective endocarditis: implications for avoiding routine transesophageal echocardiography in MRSA bacteremia

**DOI:** 10.1007/s10096-015-2539-y

**Published:** 2015-12-16

**Authors:** P. Buitron de la Vega, P. Tandon, W. Qureshi, Y. Nasr, R. Jayaprakash, S. Arshad, D. Moreno, G. Jacobsen, K. Ananthasubramaniam, M. Ramesh, M. Zervos

**Affiliations:** Division of Infectious Diseases, Henry Ford Health System, Detroit, MI USA; Wayne State University School of Medicine, Detroit, MI USA; Division of Internal Medicine, Ottawa Hospital, Ottawa, ON Canada; Government Kilpauk Medical College Hospital, Chennai, India; Heart and Vascular Institute, Henry Ford Health System, Detroit, MI USA; Division of Internal Medicine, Boston Medical Center, 801 Massachusetts Ave Crosstown, 2nd Floor, Boston, MA 02118 USA; Boston University, Boston, MA USA; Division of Cardiology, Department of Internal Medicine, Wake Forest University School of Medicine, Winston Salem, NC USA; Department of Public Health Sciences, Henry Ford Health System, Detroit, MI USA

## Abstract

The aim of this study was to identify patients with methicillin-resistant *Staphylococcus aureus* (MRSA) bacteremia with low risk of infective endocarditis (IE) who might not require routine trans-esophageal echocardiography (TEE). We retrospectively evaluated 398 patients presenting with MRSA bacteremia for the presence of the following clinical criteria: intravenous drug abuse (IVDA), long-term catheter, prolonged bacteremia, intra-cardiac device, prosthetic valve, hemodialysis dependency, vertebral/nonvertebral osteomyelitis, cardio-structural abnormality. IE was diagnosed using the modified Duke criteria. Of 398 patients with MRSA bacteremia, 26.4 % of cases were community-acquired, 56.3 % were health-care-associated, and 17.3 % were hospital-acquired. Of the group, 44 patients had definite IE, 119 had possible IE, and 235 had a rejected diagnosis. Out of 398 patients, 231 were evaluated with transthoracic echocardiography (TTE) or TEE. All 44 patients with definite IE fulfilled at least one criterion (sensitivity 100 %). Finally, a receiver operator characteristic (ROC) curve was obtained to evaluate the total risk score of our proposed criteria as a predictor of the presence of IE, and this was compared to the ROC curve of a previously proposed criteria. The area under the ROC curve for our criteria was 0.710, while the area under the ROC curve for the criteria previously proposed was 0.537 (*p* < 0.001). The *p*-value for comparing those 2 areas was less than 0.001, indicating statistical significance. Patients with MRSA bacteremia without any of our proposed clinical criteria have very low risk of developing IE and may not require routine TEE.

## Introduction

*Staphylococcus aureus* bacteremia (SAB) is a leading cause of mortality and morbidity in both nosocomial and community settings [1–5]. It is the second most common cause of hospital bloodstream infections, and has become the leading cause of infective endocarditis (IE) in most parts of the world [6–11]. Despite recent advances in diagnosis and treatment, IE remains a serious and deadly disease [8, 12–16]. Its 30-day all-cause mortality remains as high as 23.9 % in left-sided IE and 11.8 % in right-sided IE [17, 18]. The classic peripheral stigmata of IE are often nonspecific or missing, particularly among patients in whom IE is the result of *Staphylococcus aureus* infection [19, 20]. The high mortality of untreated IE, accompanied by a high prevalence of patients without clinical manifestations [8], emphasizes the importance of a diagnostic strategy sensitive enough for disease detection [21, 22]. The current European Society of Cardiology, the American College of Cardiology/American Heart Association guidelines for IE, and other recent studies recommend performing routine transthoracic echocardiography (TTE) in all patients with suspected IE [19, 23–25]. Furthermore, cost-effective calculations suggest that transesophageal echocardiography (TEE) should be done first in adults with suspected IE [19]. It has also been suggested that all patients with SAB should be considered as high risk for developing IE, and they should all undergo TTE/TEE evaluation [23, 24, 26]. Habib et al. suggested that a negative TTE in patients with SAB should be followed by TEE, due to the high clinical suspicion of IE in patients with SAB [27]. Whether all patients with SAB need a TEE is an unsettled issue. Recent literature suggests that further work is needed to identify a subgroup of patients with SAB that might only need TTE for their evaluations of IE [19]. It has been proposed that the absence of certain clinical characteristics can identify patients with SAB with low risk of IE that might not require TEE evaluation [1, 28]. As such, the aim of our study was to identify patients with SAB with low risk of IE by using simplified prediction criteria that include common risk factors for IE.

## Methods

### Hospital patients and settings

We retrospectively identified all consecutive patients with methicillin-resistant *Staphylococcus aureus* (MRSA) bacteremia diagnosed at a large tertiary care center, Henry Ford Hospital in Detroit, Michigan from 2005 to 2009. Cases of MRSA bacteremia were identified from review of the records of the clinical microbiology laboratory. All patients ≥18 years old with community-acquired, health-care-associated, or nosocomial MRSA who had ≥1 blood culture positive for MRSA were included in the study. The Henry Ford Hospital Institutional Review Board approved the study protocol.

### Data acquisition

Patients with MRSA bacteremia were evaluated for date, duration, epidemiologic source and number of positive blood cultures, source of infection, clinical signs of IE, presence of vascular events (emboli, hemorrhage), hemodialysis dependency, short-term catheter, implantable catheter, fistula or graft, diabetes, vertebral or non-vertebral osteomyelitis, prosthetic heart valve, intravenous drug use (IVDU), intra-cardiac device, cardio-structural abnormality, new conduction block, infective endocarditis diagnosis, type and timing of ultrasound studies, specialty of physician ordering the ultrasound.

### Definitions

MRSA bacteremia was defined as ≥1 positive blood culture, and it was considered (1) hospital-acquired if the blood culture was positive ≥48 h after admission and infection was not present or incubating at time of admission, (2) health-care-associated if infection was outpatient or within the first 48 h of hospitalization and the patient was hospitalized within the previous year, or (3) community-acquired if infection was outpatient or within the first 48 h of hospitalization and the patient was not hospitalized within the previous year [29]. The source of bacteremia was defined as the most likely source responsible for the first possible blood culture result on the basis of clinical signs, imaging, and microbiological findings. Prolonged bacteremia was considered “documented” if follow-up blood cultures yielded MRSA 2–4 days (at least 12 h after initial blood culture) after the first positive blood culture result. We selected this definition of prolonged bacteremia with the goal of making our criteria more sensitive to detect patients with lower risk of IE. If a patient did not have a follow-up blood culture, they were considered in the group of “possible” prolonged bacteremia. In our analysis, “possible” and “documented” bacteremia were considered as prolonged bacteremia.

Short-term catheter was defined as any peripherally inserted central catheter (PICC), internal jugular vein catheter, subclavian vein catheter, radial arterial catheter, or femoral arterial catheter or arterial line. Implantable catheter was defined as any tunneled catheter or mediport. Intra-cardiac device was defined as any implantable cardioverter–defibrillator (ICD) or permanent pacemaker (PPM). Previous IE was defined as any prior episode of IE, whether related or not related to an infected intra-cardiac device. IE was defined as definite based on modified Duke criteria [30]. IE was excluded based on modified Duke criteria [30].

### Criteria set

The criteria set was devised based on previous studies that have identified significant IE risk factors in a proposed clinical criteria including: prolonged bacteremia [31, 32], permanent intra-cardiac device (e.g., a prosthetic heart valve, pacemaker, or cardioverter–defibrillator) [33, 34], hemodialysis dependency [35], spinal infection (e.g., vertebral osteomyelitis, epidural, subdural or intra-spinal empyema, or abscess) and non-vertebral osteomyelitis [1, 36, 37]. This criteria set was found to have a sensitivity of 97.5–100 % and a negative predictive value of 92.2–100 % [1]. One of its limitations is its applicability to other populations with different risk factor profiles. Other studies have also included risk factors such as intravenous drug use [24, 38], known native valve abnormality [24, 38], previous IE [24, 38], and presence of central venous access [38–40]. Taking into consideration these previously proposed risk factors and our population profile, we developed our modified criteria for predicting IE, including prolonged bacteremia, intravenous drug use, implantable catheter, intra-cardiac device, prosthetic valve, hemodialysis, osteomyelitis, previous infective endocarditis, and cardio-structural abnormality.

Echocardiography data: all echocardiograms (TTE and TEE) were performed by the American Society of Echocardiography Level 2 and 3 trained echocardiographers. A vegetation was defined as a definite oscillating mass, irregular in character, located on the atrial surface of the mitral and tricuspid valves or ventricular surface of the aortic or pulmonic valves. If there was suspicion of vegetation, abnormal leaflet thickening, abscess, or worsening of valvular regurgitation noted on TTE, a TEE was recommended as a follow-up study. If image quality on TTE was not sufficient to assess valves and mural endocardium, a TEE was also recommended for further evaluation.

### Data analysis

The individual risk variables have been scored as 0 (no) or 1 (yes) and added together to create a total risk score. Then a cross-tabulation table was used to display the association of the total risk score with endocarditis status, with the Cochran–Armitage trend test used to evaluate the strength of that association. Furthermore, to evaluate the total risk score as a predictor of the presence of endocarditis, a receiver operating characteristic (ROC) curve was created, and the resulting area under the ROC curve was calculated. The optimal cut point from the total risk score was defined as the one containing the maximum sum of the sensitivity plus specificity for predicting endocarditis. Finally, logistic regression modeling was used to evaluate the multivariable association of endocarditis status with the individual risk variables and patient characteristics.

## Results

A total of 398 patients with MRSA bacteremia were identified and included in the analysis. Of these, 59.8 % were male, and the mean age was 58.6 ± 17.1 years: 105 (26.4 %) were community-acquired, 224 (56.3 %) were health-care-associated and 69 (17.3 %) were hospital-acquired. The median duration of the MRSA bacteremia was 4 (2 to 6) days. Of the patients in our cohort, 161 (40.5 %) had diabetes, 90 (22.6 %) were hemodialysis-dependent, 20 (5 %) had human immunodeficiency virus, 85 (21.3 %) were IVDU, and 19 (4.7 %) had a prosthetic valve. The demographic and other clinical features of the cohort are displayed in Table 1.

**Table 1:** Characteristics of 398 patients with MRSA bacteremia

Characteristics	All patients (*n* = 398)	Patients with IE (*n* = 44)	Patients without IE (*n* = 354)
Age	58.6 ± 17.1	44 ± 12.4	59.7 ± 17.3
Male	238 (59.8 %)	24 (54.5 %)	214 (60.5 %)
Nursing home resident	58 (14.6 %)	2 (4.5 %)	56 (15.8 %)
MRSA bacteremia characteristics			
Community	105 (26.4 %)	22 (50 %)	83 (23.4 %)
Health care-associated	224 (56.3 %)	18 (40.9 %)	206 (58.2 %)
Hospital-acquired	69 (17.3 %)	4 (9.1 %)	65 (18.4 %)
Duration of MRSA bacteremia (median)	4.47 days	6.86 days	4.12 days
Presumed source of infection			
Graft infection	10 (2.5 %)	1 (2.3 %)	9 (2.5 %)
Short term catheter	55 (13.8 %)	0 (0 %)	55 (15.5 %)
Implantable catheter	33 (8.3 %)	3 (6.8 %)	30 (8.5 %)
Skin/wound	132 (33.2 %)	9 (20.5 %)	123 (34.7 %)
Intra-abdominal	10 (2.5 %)	1 (2.3 %)	9 (2.5 %)
Respiratory	48 (12.1 %)	9 (20.5 %)	39 (11 %)
Para-spinal abscess	2 (0.5 %)	1 (2.3 %)	1 (0.3 %)
Urinary tract	36 (9 %)	0 (0 %)	36 (10.3 %)
Other	72 (18.1 %)	20 (45.5 %)	52 (14.7 %)
Comorbidities			
Diabetes	161 (40.5 %)	13 (29.5 %)	148 (41.8 %)
Hemodialysis	90 (22.6 %)	4 (9.1 %)	86 (24.3 %)
HIV	20 (5 %)	5 (11.4 %)	15 (4.2 %)
Nursing home resident	58 (14.6 %)	2 (4.5 %)	56 (15.8 %)

### Diagnosis of infective endocarditis

The diagnosis of IE was classified as definite in 44 [11], possible in 119 (29.9 %) and rejected in 235 (59 %) based on modified Duke criteria. Of these, 14 (3.5 %) patients had tricuspid valve vegetation, 12 (3 %) mitral valve vegetation, and eight (2 %) aortic valve vegetation. Of the total 398 patients, 241 (60.5 %) were evaluated with echocardiography (TTE or TEE). Of the 157 patients not evaluated with echocardiography, 116 (73.8 %) were categorized as rejected infective endocarditis, based on the modified Duke criteria, nine (5.7 %) had a concurrent diagnosis of osteomyelitis/cellulitis, seven (4.4 %) expired during the initial hospital stay, two (1.3 %) left against medical advice before undergoing echocardiography, and one (0.6 %) was unable to proceed with TEE due to restricted cervical motion.

Of the total 241 patients evaluated with echocardiography, 114 (47.3 %) were evaluated with TTE only, 56 (23.2 %) TEE only, and 71 (29.5 %) TTE followed by TEE. Of the 241 patients evaluated with echocardiography studies, 234 (97.1 %) had their initial study done within 14 days after the first positive blood culture result. Of the total 44 patients with definite IE diagnosis, 24 (54.5 %) were evaluated with both TTE and TEE, ten (22.7 %) were evaluated with TEE, and ten (22.7 %) were evaluated with TTE alone.

### Prediction criteria for diagnosis of IE

The significant clinical predictors for diagnosis of IE are provided in Table 2. We considered less than one positive variable as predicting low risk of IE. In total, 44 patients with documented IE fulfilled at least one criterion (sensitivity 100 %, negative predictive value 100 %, specificity 19.2 %, positive predictive value 13.3 %). Of the 44 patients with documented IE, 34 fulfilled at least two criteria (sensitivity 77.3 %, negative predictive value 95.2 %, specificity 55.6 %, and positive predictive value 17.8 %). Among the 68 patients that fulfilled less than one of the clinical predictors, 23 (33.8 %) were evaluated with TTE as the first imaging study, five (7.4 %) with TEE alone, one (1.5 %) underwent TEE followed by TTE, and 39 (57.5 %) did not have any imaging done. Of the 39 patients not evaluated with echocardiography, 33 (86.4 %) were categorized as rejected IE based on the modified Duke criteria, four (10.3 %) expired during the initial hospital stay, and in two cases (5.1 %), there was not enough information in the chart review to determine the reason for not evaluating with imaging.

**Table 2:** Clinical prediction criteria associated with increased risk of IE in patients with MRSA bacteremia

Prediction criterion	All patients (*n* = 398)	Without IE (*n* = 354)	With IE (*n* = 44)	*P*-value	Odds ratio	Odds ratio 95 % confidence limits	
Prolonged bacteremia	269 (67.5 %)	232 (65.5 %)	37 (84.1 %)	0.167	2.131	0.729	6.229
Intravenous drug use	85 (21.3 %)	53 (15 %)	32 (72.7 %)	<0.001*	14.951	5.356	41.734
Implantable catheter	60 (15 %)	57 (16.1 %)	3 (6.8 %)	0.387	3.441	0.209	56.657
Intracardiac device	19 (4.7 %)	18 (5.1 %)	1 (2.3 %)	0.384	0.280	0.016	4.917
Prosthetic valve	19 (4.7 %)	7 (2 %)	6 (13.6 %)	0.002*	13.086	2.529	67.704
Hemodialysis	90 (22.6 %)	86 (24.3 %)	4 (9.1 %)	0.122	0.068	0.002	2.040
Osteomyelitis	52 (13 %)	46 (13 %)	6 (13.6 %)	0.067	0.287	0.076	1.089
Previous IE	19 (4.7 %%)	10 (2.8 %)	9 (20.5 %)	0.117	2.849	0.769	10.554
Cardio-structural abnormality	13 (3.2 %)	9 (2.5 %)	4 (9.1 %)	0.035*	6.095	1.132	32.833

*Statistically significant, *P* < 0.05

**Fig. 1:**
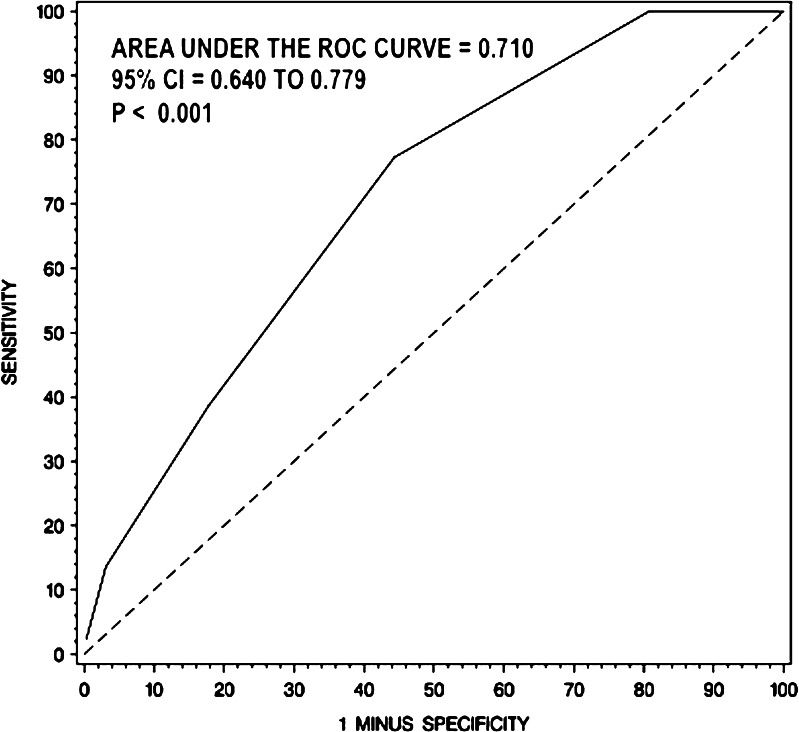
ROC curve for our proposed clinical prediction criteria set

**Fig. 2:**
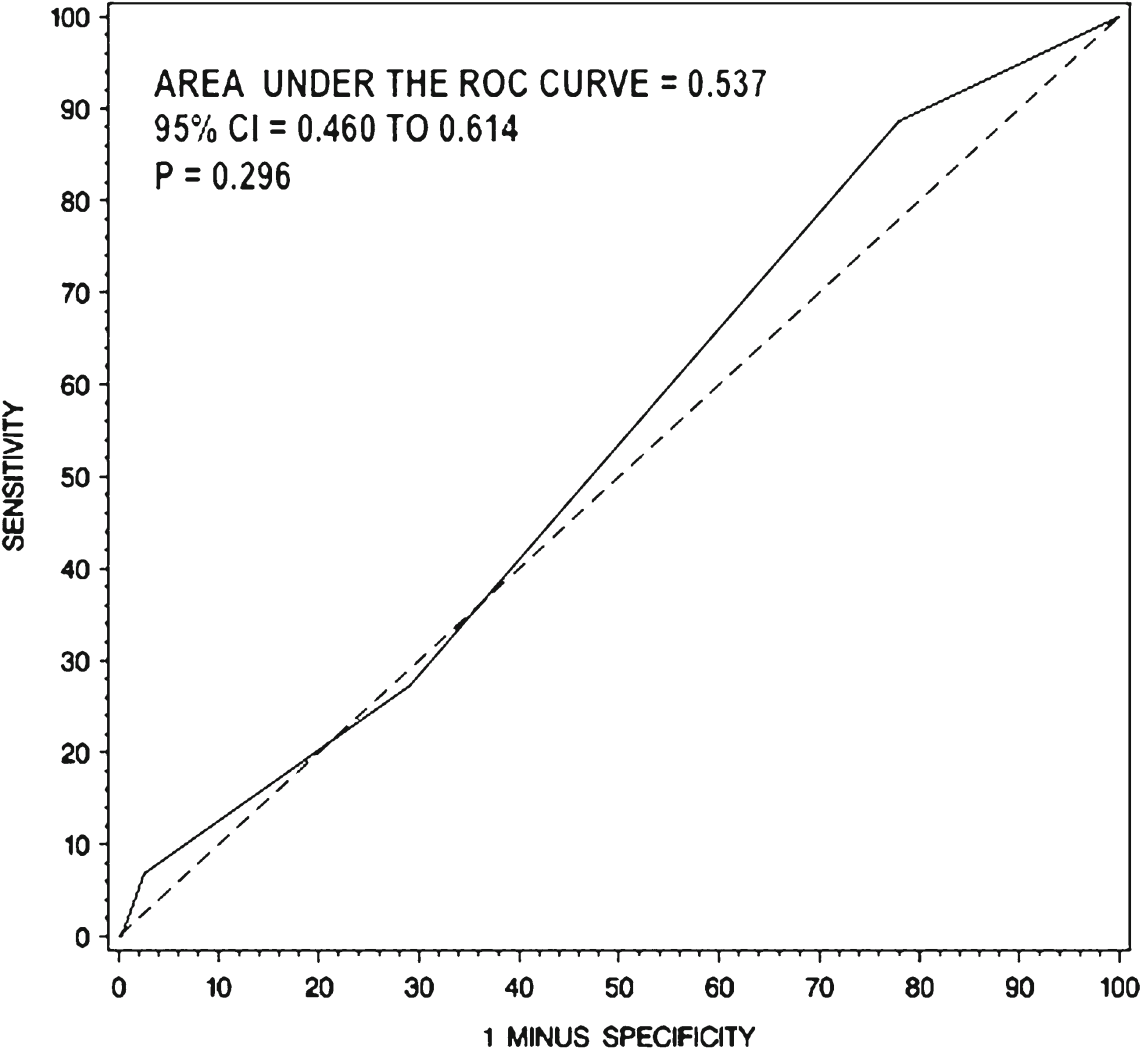
ROC curve for clinical prediction criteria set proposed by Kaasch et al. [1]

A ROC curve was obtained using our criteria (Fig. 1) and was compared to the criteria proposed by Kaasch et al. [1] (Fig. 2), which included prolonged bacteremia, permanent intracardiac device, prosthetic heart valve, pacemaker, cardioverter–defibrillator, hemodialysis dependency, spinal infection, or non-vertebral osteomyelitis. The area under the ROC curve for our criteria was 0.710 (*p* < 0.001), while the area under the ROC curve for the simple criteria proposed by Kaasch et al. [1] was 0.537 (*p* = 0.296). When comparing the ROC curve areas to a reference area of 0.50 (the area that would be expected if no predictive ability existed), our criteria had significantly better ability to predict IE than Kaasch et al. [1] (*P* < 0.001).

### Association of the prediction criteria with mortality

When applying our criteria to predict mortality, we determined that 19 (27.9 %) of the 68 patients with less than one of the proposed criteria expired within 30 days of their diagnosis of MRSA bacteremia, compared to 68 (20.6 %) of the 330 patients with one or more than one of the proposed criteria. We found a sensitivity of 78.2 %, a specificity of 15.8 %, a positive predictive value of 20.6 % and a negative predictive value of 72.1 % with a chi-square test *p*-value of 0.183, demonstrating no statistically significant association of our proposed prediction criteria with mortality. When applying the criteria proposed by Kaasch et al. [1], we found that 19 (22.9 %) of the 83 patients with less than one of their proposed criteria died within 30 days of their diagnosis, compared to 68 (27.5 %) of the 247 patients with one or more than one of the proposed criteria. We found a sensitivity of 78.2 %, specificity of 20.6 %, positive predictive value of 21.6 % and a negative predictive value of 77.1 with a chi-square test *p*-value of 0.798, also showing no statistically significant association of their proposed prediction criteria with mortality.

## Discussion

Our study accurately demonstrated that our simplified criteria to risk stratify SAB patients effectively predicted patients with MRSA bacteremia with very low risk of developing IE. Previous analysis by Kaasch et al. also demonstrated a high sensitivity to diagnose IE by using simplified criteria [1], although when the same criteria set was applied to our cohort population, the area under the curve was smaller than our proposed criteria that also included implantable catheter, IVDA, cardio-structural abnormality and previous IE. These four extra criteria added in our study represent important risk factors in an inner-city population with high prevalence of IVDU and implantable catheters.

In this retrospective analysis of patients with MRSA bacteremia, 44 (11 %) were diagnosed with infective endocarditis. This prevalence of endocarditis in SAB is similar to previous studies that have reported values between 5 and 17 % [1, 41–43,] and other studies have reported values up to 32 % [24, 44, 45]. The 44 patients with IE had a 30-day overall mortality of 25 % that is comparable to other reported studies ranging from 13 to 31 % mortality [2, 41].

It is interesting to point out the lack of association of our criteria with mortality. One of the possible explanations of this result could be the fact that patients with more risk factors are provided with advanced treatment protocols and early empirical antimicrobial therapy. As has been previously reported, inadequacy of empirical antimicrobial therapy in these patients is an important risk factor for mortality [46]. It has also been reported that the APACHE II score has been associated with mortality in patients with MRSA bacteremia [47]. Most of the included variables in the APACHE II are not included in our prediction criteria, as they mainly measure hemodynamic values of the patients that are not necessary associated with IE.

### Clinical implications

Although the current European Society of Cardiology, the ACC/AHA guidelines and other recent studies recommend performing routine echocardiography in all patients with SAB [19, 23, 24], only 241 (60.5 %) of the cases in our study complied with this recommendation, being one of the highest reported [48]. Other studies have shown compliance results ranging from 39.9 to 73 % [1, 24, 49, 50]. Even in a cohort of patients with SAB presenting with intra-cardiac device and higher risk of endocarditis, echocardiography was obtained in only 77 % of cases [51]. An accurate definition of low probability of IE in patients with SAB is still lacking, and is necessary to provide better guidance of how to use echocardiography. Our criteria could potentially be used as a tool to identify patients with MRSA bacteremia at low risk of endocarditis, in order to guide the mode of echocardiography. In patients with one or more of the proposed criteria, the risk of developing IE is higher, and TEE should be considered in their evaluation. In patients with less than one of the proposed criteria, their risk of IE is low and TEE might not be needed for their evaluation. In our cohort, TEE results did not change or add to the management in any of the low-risk patients. It could be argued that in patients with community-acquired MRSA and very low risk of IE, the real duration of bacteremia is unknown, and as such, considering ordering a TTE should not be discouraged. While patients with less than one of the proposed criteria are at low risk of IE, they are still at very high risk of poor outcomes. Absence of IE does not imply absence of complicated disease in patients with MRSA bacteremia. It will be important to validate these criteria prospectively and follow the subjects in the long term in order to include missed cases of endocarditis.

## Limitations

As our analysis was retrospective, there are several inherent limitations in this type of study design. Adequately identifying missed cases of IE was not possible, as there was no follow-up arranged. With the goal of capturing patients with missed IE, we performed a second chart review of medical records that corresponded to 6 months after MRSA was initially diagnosed. Even though we did not find any information supporting missed IE in patients with none of our prediction criteria, the review was still limited by the fact that we did not capture the actual cause of death, so some patients could have followed up at another institution or died before having a complete evaluation for IE. It is important to mention that source control was at discretion of the clinicians caring for the patient; as a retrospective chart review, this information was not consistently available. Additionally, not all patients in our cohort underwent TTE or TEE evaluation, limiting the diagnostic power of Duke criteria due to the fact that IE is not easily recognized by physical examination. This also limited our ability to classify patients as rejected IE. We performed a second chart review of all 116 patients that were classified as rejected IE, with the goal of identifying the reasons why no imaging was done. All of these 116 patients either had a firm alternate diagnosis for the manifestations of IE, the manifestations of infection resolved within 4 days, or they did not meet criteria for possible IE. The use of Duke criteria in these patients continues to be limited due to the fact that we did not have echocardiography evaluations or pathologic evidence at surgery or autopsy from any of them. As such, the actual number of IE cases may have been underestimated.

## Conclusion

In conclusion, patients with MRSA bacteremia and at least one of our proposed clinical criteria have an inherently higher risk of IE, and TEE should be considered for their evaluation. Patients with less than one of our proposed criteria have very low risk of developing IE and might not need TEE for their evaluation. It is important to mention that in patients with less than one of our proposed criteria, physicians should not be discouraged from performing TTE in their evaluation, as such patients are still considered at risk of developing IE.

## Abbreviations

IE: Infective endocarditis
ICD: Implantable cardioverter-defibrillator
IVDA: Intravenous drug abuse
PICC: Peripherally inserted central catheter
MRSA: Methicillin-resistant Staphylococcus aureus
ROC: Receiver operator characteristic
SAB: Staphylococcus aureus bacteremia
TEE: Trans-esophageal echocardiography
TTE: Trans-thoracic echocardiography
